# Fucoxanthin Ameliorates MASLD by Directly Targeting GRP78 to Restore ER Homeostasis and Activate AMPK Signaling

**DOI:** 10.1002/fsn3.71813

**Published:** 2026-04-30

**Authors:** Lin Zhang, Ruifen Zhang, Yu Wu, Binbin Chen, Chao Wang, Cheng Wang, Zhengshun Wen, Jingui Zhang, Haibin Tong, Liang Hong

**Affiliations:** ^1^ Department of Pharmacy Qionghai People's Hospital Qionghai China; ^2^ Zhejiang Provincial Key Laboratory for Water Environment and Marine Biological Resources Protection, College of Life and Environmental Science Wenzhou University Wenzhou China; ^3^ Xianghu Laboratory Hangzhou China; ^4^ Phase I Clinical Trial Ward, Hainan General Hospital Hainan Affiliated Hospital of Hainan Medical University Haikou China; ^5^ State Key Laboratory for Quality Ensurance and Sustainable Use of Dao‐di Herbs Beijing China; ^6^ Department of Infectious Diseases The Third Affiliated Hospital of Wenzhou Medical University Wenzhou China

**Keywords:** AMP‐activated protein kinase, endoplasmic reticulum stress, fucoxanthin, glucose‐regulated protein 78, metabolic associated fatty liver disease

## Abstract

The identification of direct molecular targets for bioactive dietary components is critical for precision nutrition intervention in metabolic dysfunction‐associated steatotic liver disease (MASLD). Fucoxanthin, a marine carotenoid from *Sargassum fusiforme*, exhibits potent lipid‐lowering effects; however, its precise intracellular targets and upstream regulatory mechanisms remain elusive. Herein, using drug affinity responsive target stability (DARTS) coupled with LC–MS/MS, we identified the endoplasmic reticulum (ER) chaperone glucose‐regulated protein 78 (GRP78) as a direct binding target of fucoxanthin. Molecular dynamics (MD) simulations and cellular thermal shift assays (CETSA) confirmed a stable interaction, primarily driven by hydrogen bonding at the ARG74 residue. In *ob/ob* mice and palmitic acid‐induced HepG2 cells, fucoxanthin treatment significantly alleviated hepatic steatosis and suppressed ER stress. Mechanistically, the fucoxanthin‐GRP78 interaction was found to be indispensable for the subsequent activation of AMP‐activated protein kinase (AMPK) signaling. Notably, siRNA‐mediated knockdown of GRP78 or pharmacological inhibition of AMPK completely abolished the lipid‐lowering and ER stress‐relieving effects of fucoxanthin, confirming a causal GRP78‐AMPK axis. This study elucidates a novel target‐driven mechanism wherein fucoxanthin acts as a GRP78 ligand to restore ER homeostasis and reprogram lipid metabolism. These findings position the fucoxanthin‐GRP78 axis as a specific therapeutic target for nutritional strategies against MASLD.

## Graphical abstract

Proposed model of fucoxanthin in ameliorating MASLD via the GRP78‐AMPK axis. In MASLD, excessive lipid accumulation (FFA, TG, DNL) leads to ER stress activation (upregulation of CHOP, ATF6, IRE1) and GRP78 dysfunction, promoting hepatic lipid droplet (LD) formation. Fucoxanthin directly binds to GRP78, which in turn activates AMPK signaling. Activated AMPK inhibits Srebf1c‐mediated de novo lipogenesis (DNL), thereby reducing lipid synthesis, alleviating ER stress (as indicated by decreased CHOP and ATF6), and attenuating hepatocyte lipid droplet accumulation. This fucoxanthin‐GRP78‐AMPK cascade restores ER homeostasis and reprograms lipid metabolism, ultimately ameliorating MASLD progression.

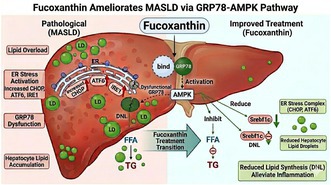

## 1 Introduction

Over the past decade, shifts in global lifestyle and dietary patterns have fueled a dramatic rise in the prevalence of metabolic dysfunction‐associated steatotic liver disease (MASLD, formerly known as NAFLD/MAFLD) (Yoon et al. 2021). Currently affecting approximately 38% of the adult population worldwide, MASLD has emerged as the most common chronic liver disease (Chan et al. 2023; Younossi et al. 2025). The pathology initiates with hepatic steatosis—an imbalance in lipid metabolism—and, if left unmanaged, can progress to metabolic dysfunction‐associated steatohepatitis (MASH), fibrosis, and eventually hepatocellular carcinoma (Huang, Wong, et al. 2025; Kim et al. 2023). Despite this growing health burden, approved pharmacological therapies that specifically target the core pathological feature of MASLD—hepatic lipid accumulation—remain scarce, with only resmetirom recently approved for a subset of patients with advanced fibrosis (Noureddin et al. 2024; Wu et al. 2025). While lifestyle interventions remain the cornerstone of management, long‐term adherence is often poor. Consequently, there is an urgent demand to identify bioactive dietary components capable of resolving hepatic lipid deposition to halt or reverse disease progression.

Dysfunction of the Endoplasmic Reticulum (ER), the central organelle governing secretory protein folding, lipid biogenesis, and calcium homeostasis, acts as a primary driver of MASLD pathologies (Venkatesan et al. 2023). In hepatocytes, persistent disturbances—particularly the excessive influx of free fatty acids (FFAs) associated with obesity—precipitate a state of chronic ER stress (Bansal and Bansal 2024). To counter this, the ER engages an evolutionarily conserved pathway termed the unfolded protein response (UPR) to restore homeostasis by enhancing protein folding capacity and promoting autophagy‐dependent clearance (Christianson et al. 2011; Hotamisligil 2010). However, in the context of severe steatosis, these protective mechanisms are rapidly overwhelmed. This maladaptive UPR fuels a “vicious cycle” of disease progression by inducing de novo lipogenesis (DNL), altering mitochondrial activity, and impairing very‐low‐density lipoprotein (VLDL) trafficking (Celik et al. 2023; Song and Malhi 2019). Crucially, the ER chaperone glucose‐regulated protein 78 (GRP78) sits at the apex of this regulatory network. Under physiological conditions, GRP78 acts as a gatekeeper by binding to transmembrane stress sensors (IRE1, PERK, ATF6) to maintain their quiescence. Upon lipid overload, GRP78 dissociates to handle misfolded proteins, thereby triggering UPR signaling (Kettel and Karagöz 2024; Shore et al. 2011). Consequently, GRP78 dysfunction is intrinsically linked to the dysregulation of hepatic lipid metabolism (He et al. 2025; Luo et al. 2022). Therefore, pharmacological strategies aimed at modulating GRP78 to restore ER homeostasis represent a promising approach to break the link between metabolic overload and hepatic injury.

The ocean serves as a vast reservoir of natural bioactive compounds with metabolic regulatory potential (Wu et al. 2023). Fucoxanthin, a marine carotenoid abundant in brown algae such as *Sargassum fusiforme*, has demonstrated remarkable efficacy in combating obesity and insulin resistance (Li et al. 2020; Wu et al. 2024; Yusof et al. 2022). Our previous investigations revealed that fucoxanthin alleviates hepatic steatosis by activating the AMP‐activated protein kinase (AMPK) signaling pathway (Yang et al. 2024), a master sensor of cellular energy status. However, a critical knowledge gap remains regarding the precise molecular initiation of this event. As AMPK is a cytosolic kinase complex, the mechanism by which the lipophilic fucoxanthin molecule triggers its activation remains elusive. Specifically, the direct upstream cellular targets that physically interact with fucoxanthin to transduce its lipid‐lowering signals have not been identified, limiting the understanding of its pharmacological mode of action.

In this study, we employed an integrated approach combining bioinformatics, chemical biology, and molecular simulation to elucidate the direct mechanism of action of fucoxanthin. First, transcriptome analysis of clinical datasets highlighted the convergence of ER stress and AMPK signaling as pivotal nodes in MASLD pathogenesis. Guided by these findings, we utilized drug affinity responsive target stability (DARTS) coupled with mass spectrometry—a label‐free target engagement strategy (Huang, Zhang, et al. 2025)—to screen for direct binding partners. We identified the ER chaperone GRP78 as a primary physical target of fucoxanthin. Herein, we provide the first evidence that fucoxanthin functions as a functional ligand of GRP78. We demonstrate that this specific interaction stabilizes ER function and initiates a beneficial GRP78‐AMPK signaling axis, thereby ameliorating hepatic steatosis. This work not only elucidates the target‐driven mechanism of fucoxanthin but also positions the GRP78‐AMPK axis as a viable target for nutritional intervention in liver diseases.

## 2 Materials and Methods

### 2.1 Materials and Chemicals

Fucoxanthin (purity ≥ 95%) was isolated and purified from *Sargassum fusiforme* and provided by Ningbo Tianhong Biotech Co. Ltd. (Ningbo, China). Palmitic acid (PA, #P5585), Bovine Serum Albumin (BSA), and protease inhibitor cocktail (#P8340) were purchased from Sigma‐Aldrich (St. Louis, MO, USA). Dulbecco's Modified Eagle's Medium (DMEM) and fetal bovine serum (FBS) were obtained from Gibco (Grand Island, NY, USA). Pronase (#10165921001) was purchased from Roche Diagnostics (Mannheim, Germany). Pierce BCA Protein Assay Kit (#A55864) was purchased from Thermo Fisher Scientific (Waltham, MA, USA). Primary antibodies against GRP78 (#3177), eIF2α (#9722), and phospho‐eIF2α (Ser51, #3398) were purchased from Cell Signaling Technology (Beverly, MA, USA). Antibodies against AMPKα (#ab32047) and phospho‐AMPKα (Thr172, #ab133448) were obtained from Abcam (Cambridge, UK). The antibody against Tubulin (#sc‐47,724) and HRP‐conjugated secondary antibodies were purchased from Santa Cruz Biotechnology (Santa Cruz, CA, USA). All other chemical reagents used were of analytical grade.

### 2.2 Bioinformatic Analysis of MASLD‐Related Datasets

Transcriptomic datasets comparing liver tissue from MASLD patients and healthy controls were retrieved from the Gene Expression Omnibus (GEO) database (Accession No. GSE48452). Differentially Expressed Genes (DEGs) were identified using the GEO2R tool with a threshold of |log2 Fold Change| > 1 and adjusted *P*‐value < 0.05. Functional annotation was performed using the DAVID database (v6.8) for Gene Ontology (GO) and Kyoto Encyclopedia of Genes and Genomes (KEGG) pathway enrichment analyses. Gene Set Enrichment Analysis (GSEA) was conducted to identify significantly enriched biological pathways, with statistical significance defined as a normalized enrichment score (|NES|) > 1 and a false discovery rate (FDR) < 0.05.

### 2.3 Cell Culture and Treatments

HepG2 human hepatoma cells were obtained from the American Type Culture Collection (ATCC, #HB‐8065) and cultured in DMEM supplemented with 10% (v/v) FBS and 1% penicillin/streptomycin in a humidified atmosphere containing 5% CO_2_ at 37°C. For lipid loading, a PA stock solution (100 mM in DMSO) was conjugated with fatty acid‐free BSA (molar ratio 6:1) to prepare a working solution. Cells were treated with 100 μM PA in the presence or absence of fucoxanthin at indicated concentrations for 24 h. Compound C (an AMPK inhibitor) or Thapsigargin (Tg, an ER stress inducer) were used as pharmacological tools where indicated.

### 2.4 Drug Affinity Responsive Target Stability (DARTS) Assay

The DARTS assay was performed to identify direct protein targets of fucoxanthin as previously described (Jia et al. 2024; Ren et al. 2021) with specific modifications to optimize target detection. Briefly, HepG2 cells were lysed in M‐PER buffer supplemented with protease and phosphatase inhibitors. The lysates were clarified by centrifugation (12,000 × *g*, 15 min, 4°C), and the protein concentration was determined using a BCA Protein Assay Kit (adjusting the final concentration to 2.5–5 μg/μL).

To determine the optimal fucoxanthin concentration for target engagement, a pilot experiment was conducted using a range of concentrations (0, 50, 100, 200, 500, and 1000 μM). The concentration of 100 μM was selected for subsequent experiments as it yielded the most distinct protected band corresponding to GRP78 without inducing non‐specific binding or oversaturation. Cell lysates were divided into equal aliquots and incubated with fucoxanthin (final concentration 100 μM) or DMSO (vehicle control) for 1 h at room temperature with continuous agitation using a thermomixer to facilitate ligand‐protein binding. To determine the optimal proteolysis conditions, a pilot experiment was conducted using a range of Pronase‐to‐protein ratios (1:100, 1:500, 1:1000, and 1:2000, w/w). A ratio of 1:100 (w/w) was selected for the final assay as it produced a consistent digestion pattern while effectively preserving the protected bands. Subsequently, samples were subjected to limited proteolysis with the optimized Pronase concentration for 20 min at room temperature. Digestion was terminated by the addition of protease inhibitors followed by 5 × SDS‐PAGE loading buffer and boiling at 100°C for 10 min. Samples were separated by SDS‐PAGE and visualized by Silver staining to observe the differential bands.

### 2.5 LC–MS/MS Analysis

The protected protein bands from DARTS proteolysis in the fucoxanthin‐treated group were excised, in‐gel digested with trypsin, and analyzed by LC–MS/MS at Novogene Co. Ltd. (Beijing, China) using an EASY‐nLC 1200 coupled to a Q Exactive HF‐X mass spectrometer. Raw data were searched against the UniProt human database using Proteome Discoverer (Sequest HT) with FDR < 1%. Proteins with ≥ 2 unique peptides were considered. Candidates were ranked by peptide score and coverage; GRP78 was selected as the top hit based on its highest score and relevance to ER stress pathways identified in bioinformatics analysis.

### 2.6 Cellular Thermal Shift Assay (CETSA)

To validate target engagement in live cells, CETSA was performed based on the principle of ligand‐induced thermal stabilization. HepG2 cells were treated with fucoxanthin (100 μM) or DMSO for 1 h. Cells were harvested, washed with PBS, and resuspended in lysis buffer supplemented with protease inhibitors. The cell suspensions were divided into aliquots and heated individually at a temperature gradient (37°C–60°C) for 3 min in a thermal cycler, followed by cooling at room temperature for 3 min. For the Isothermal Dose–Response (ITDR) CETSA, cell lysates were incubated with varying concentrations of fucoxanthin at a fixed temperature (52°C). The heated lysates were centrifuged at 20,000 × *g* for 20 min at 4°C to pellet precipitated proteins. The soluble supernatants were analyzed by Western blotting using the anti‐GRP78 antibody.

### 2.7 Molecular Docking and Molecular Dynamics (MD) Simulation

The crystal structure of the human GRP78 protein was retrieved from the Protein Data Bank (PDB) or modeled using AlphaFold 3.0 where high‐resolution crystal structures of specific domains were unavailable. Molecular docking was performed using AutoDock Vina (v1.2.6) to predict the binding pose and affinity. The complex with the lowest binding energy was selected for MD simulation.

MD simulations were carried out using GROMACS (version 2022.4) with the Amber99sb‐ildn force field for the protein and the General Amber Force Field (GAFF) for fucoxanthin. The complex was solvated in a cubic box with TIP3P water molecules, and the system was neutralized by adding Na^+^ and Cl^−^ ions. Following energy minimization (steepest descent algorithm) and equilibration (NVT and NPT ensembles for 100 ps each), a 100 ns production run was performed at 300 K and 1 bar. Trajectory analyses, including Root Mean Square Deviation (RMSD), Root Mean Square Fluctuation (RMSF), Radius of Gyration (Rg), Solvent Accessible Surface Area (SASA), and hydrogen bond occupancy, were computed. Binding free energy was calculated using the Molecular Mechanics/Poisson‐Boltzmann Surface Area (MM/PBSA) method based on the stable trajectory (last 35 ns).

### 2.8 Animal Experiments

Male *ob/ob* mice (B6.Cg‐Lepob/J, 6 weeks old) and wild‐type C57BL/6J littermates were purchased from Vital River Laboratory Animal Technology Co. Ltd. (Beijing, China). Mice were housed under specific pathogen‐free (SPF) conditions with a 12‐h light/dark cycle. After 1 week of acclimatization, *ob/ob* mice were randomly divided into two groups (*n* = 8 per group): the model group fed a standard chow diet and the treatment group fed a standard chow diet supplemented with 0.2% (w/w) fucoxanthin. Wild‐type mice served as the normal control. After 8 weeks of intervention, mice were euthanized, and liver tissues were collected, snap‐frozen in liquid nitrogen. All animal procedures were approved by the Animal Care and Use Committee of Wenzhou University (Approval no. WZU‐2023‐030).

### 2.9 Biochemical Analysis

Intracellular triglyceride (TG) was measured using enzymatic kits (Jiancheng Bioengineering Institute, Nanjing, China) according to the manufacturer's instructions.

### 2.10 Oil Red O Staining in HepG2 Cells

For evaluating lipid accumulation in HepG2 cells, the cells were fixed in paraformaldehyde for 10 min and then treated with propylene glycol for another 10 min. Following fixation, the cells were stained with oil red O solution for 10 min. Images were captured using a Leica DM300 LED microscope, and the oil red O staining area was semi‐quantitatively analyzed using the Image Pro Plus Software.

### 2.11 siRNA Transfection

Small interfering RNA (siRNA) targeting human GRP78 (sc‐44261) and a non‐targeting scramble control were purchased from Santa Cruz Biotechnology. HepG2 cells were transfected with siRNA using Lipofectamine RNAiMAX (Invitrogen) following the manufacturer's protocol. Knockdown efficiency was verified by Western blotting 48 h post‐transfection prior to subsequent fucoxanthin treatment.

### 2.12 Quantitative Real‐Time PCR (qRT‐PCR)

Total RNA was extracted using TRIzol reagent (Invitrogen). cDNA synthesis was performed using the PrimeScript RT Reagent Kit (TaKaRa, Japan). qRT‐PCR was conducted on a real‐time PCR system using SYBR Premix Ex Taq (TaKaRa). Relative gene expression levels were calculated using the2^−ΔΔct^ method, with β‐actin as the internal reference. Primer sequences are listed in Table 1.

**TABLE 1:** Designed primer sets for qRT‐PCR.

Gene	Forward primer (5′ to 3′)	Reverse primer (5′ to 3′)
Mouse		
*Ddit3*	TTAAAGATGAGCGGGTGGCA	ACTTCCTTCTTGAACACTCTCT
*Ern1*	TCTTGGGCGAACAGAATACACC	GGCCGCATAGTCAAAGTAGGT
*β‐Actin*	CATTGCTGACAGGATGCAGAAGG	TGCTGGAAGGTGGACAGTGAGG
Human		
*PPARα*	CATCCCAGGCTTCGCAAACT	TCCATACGCTACCAGCATCC
*CPT1*	GTTACGACAGGTGGTTTGAC	CGTTTGCCAGAAGATTTGCG
*SREBP1C*	GTGCTCTGCGAGTGGATG	CAGGTTGGTGGCAGTGAG
*FASN*	CGTTTGCCAGAAGATTTGCG	GGACAGAGCAACTACGGCTT
*HSPA5*	TCGATACTGGCCGAGACAAC	CGACGGTTCTGGTCTCACA
*ATF6*	GCTCTGGAACAGGGCTCAAA	ACTCCCTGAGTTCCTGCTGA
*DDIT3*	TTGAAGATGAGCGGGTGGCAG	CACGTGGACCAGGTTCTCTCT
*ERN1*	CCAGATGAAGACTGGGAGTCG	TGTCTGAGCAGAAGTGGCTG
*β‐Actin*	TGGATCAGCAAGCAGGAGTA	TCGGCCACATTGTGAACTTT

### 2.13 Western Blot Analysis

Protein lysates were prepared in RIPA buffer supplemented with protease and phosphatase inhibitors. Equal amounts of protein (30–50 μg) were separated by SDS‐PAGE and transferred onto PVDF membranes (Millipore). Membranes were blocked with 5% non‐fat milk and incubated with specific primary antibodies overnight at 4°C, followed by HRP‐conjugated secondary antibodies. Protein bands were visualized using an Enhanced Chemiluminescence (ECL) kit and quantified using ImageJ software.

### 2.14 Statistical Analysis

All data are presented as mean ± standard deviation (SD) from at least three independent experiments. Statistical differences were analyzed using GraphPad Prism 8.0 software. Comparisons between two groups were performed using the unpaired Student's *t*‐test. Multiple group comparisons were conducted using one‐way analysis of variance (ANOVA) followed by Tukey's post hoc test. A *p*‐value < 0.05 was considered statistically significant.

## 3 Results

### 3.1 Transcriptomic Analysis Reveals ER Stress and AMPK Signaling as Pivotal Pathogenic Nodes in MASLD

**FIGURE 1:**
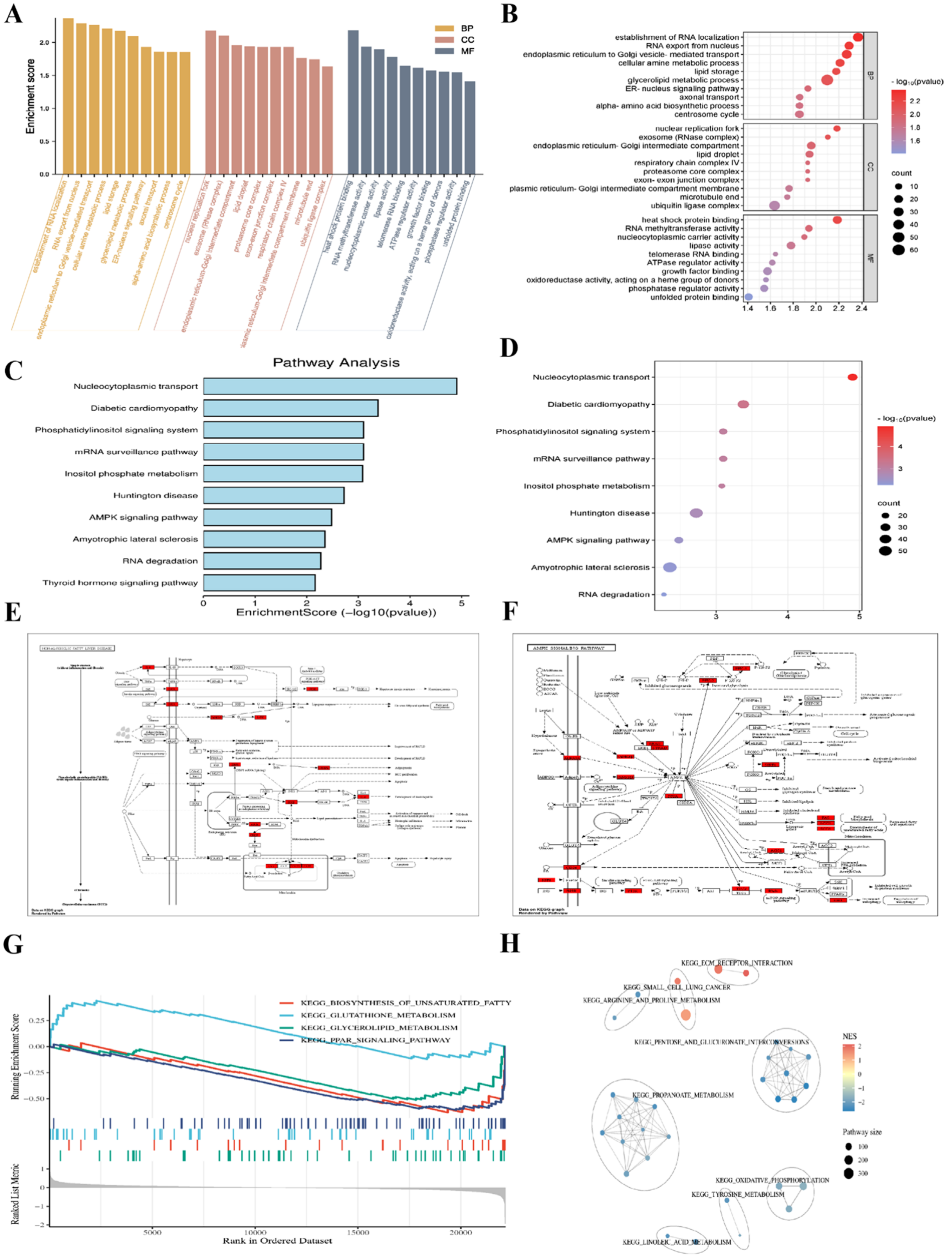
Bioinformatics analysis identifies ER stress and AMPK signaling as pivotal pathways in MASLD. (A, B) Gene ontology (GO) enrichment analysis of differentially expressed genes (DEGs) in MASLD patients versus healthy controls (GSE48452), highlighting biological processes (BP), cellular components (CC), and molecular functions (MF) related to lipid metabolism and ER stress. (C, D) KEGG pathway enrichment analysis showing significant enrichment of the AMPK signaling pathway and protein processing in the endoplasmic reticulum. (E) Pathway map of NAFLD (MASLD) pathogenesis, indicating multiple hit targets. (F) Pathway map of the AMPK signaling pathway, highlighting the enriched key nodes in MASLD. (G, H) Gene set enrichment analysis (GSEA) plots demonstrating the enrichment of gene sets related to unsaturated fatty acid biosynthesis, PPAR signaling (G), and oxidative phosphorylation (H).

To systematically decipher the molecular mechanisms underlying MASLD, we performed a comprehensive bioinformatic analysis of the GEO dataset GSE48452. Differential expression analysis identified a distinct transcriptomic profile in MASLD patients compared to healthy controls. Gene Ontology (GO) enrichment analysis revealed that the differentially expressed genes (DEGs) were predominantly enriched in biological processes related to lipid metabolism (e.g., lipid storage, glycerolipid metabolic process) and endoplasmic reticulum (ER) stress responses (e.g., response to endoplasmic reticulum stress, unfolded protein binding) (Figure 1A,B). Consistent with these findings, KEGG pathway analysis highlighted significant alterations in metabolic pathways, including the AMPK signaling pathway, NAFLD, and protein processing in the endoplasmic reticulum (Figure 1C,D). Detailed pathway mapping further visualized the suppression of AMPK signaling (Figure 1E,F) and the activation of UPR components in MASLD livers. Moreover, Gene Set Enrichment Analysis (GSEA) confirmed that gene sets related to lipid biosynthesis and PPAR signaling were significantly enriched, while oxidative phosphorylation was suppressed (Figure 1G,H). Collectively, these transcriptomic data suggest that dysregulated ER homeostasis and impaired AMPK signaling are pivotal pathogenic nodes in MASLD, guiding our subsequent search for molecular targets. To experimentally validate these bioinformatic findings, we examined the expression patterns of key markers in our experimental models. Notably, the upregulated trends of *HSPA5* (GRP78), *DDIT3* (CHOP), and FASN observed in both *ob/ob* mice and PA‐treated HepG2 cells were consistent with the human MASLD dataset (GSE48452), thereby reinforcing the translational relevance of our subsequent mechanistic investigations.

### 3.2 Identification and Structural Characterization of GRP78 as a Direct Cellular Target of Fucoxanthin

**FIGURE 2:**
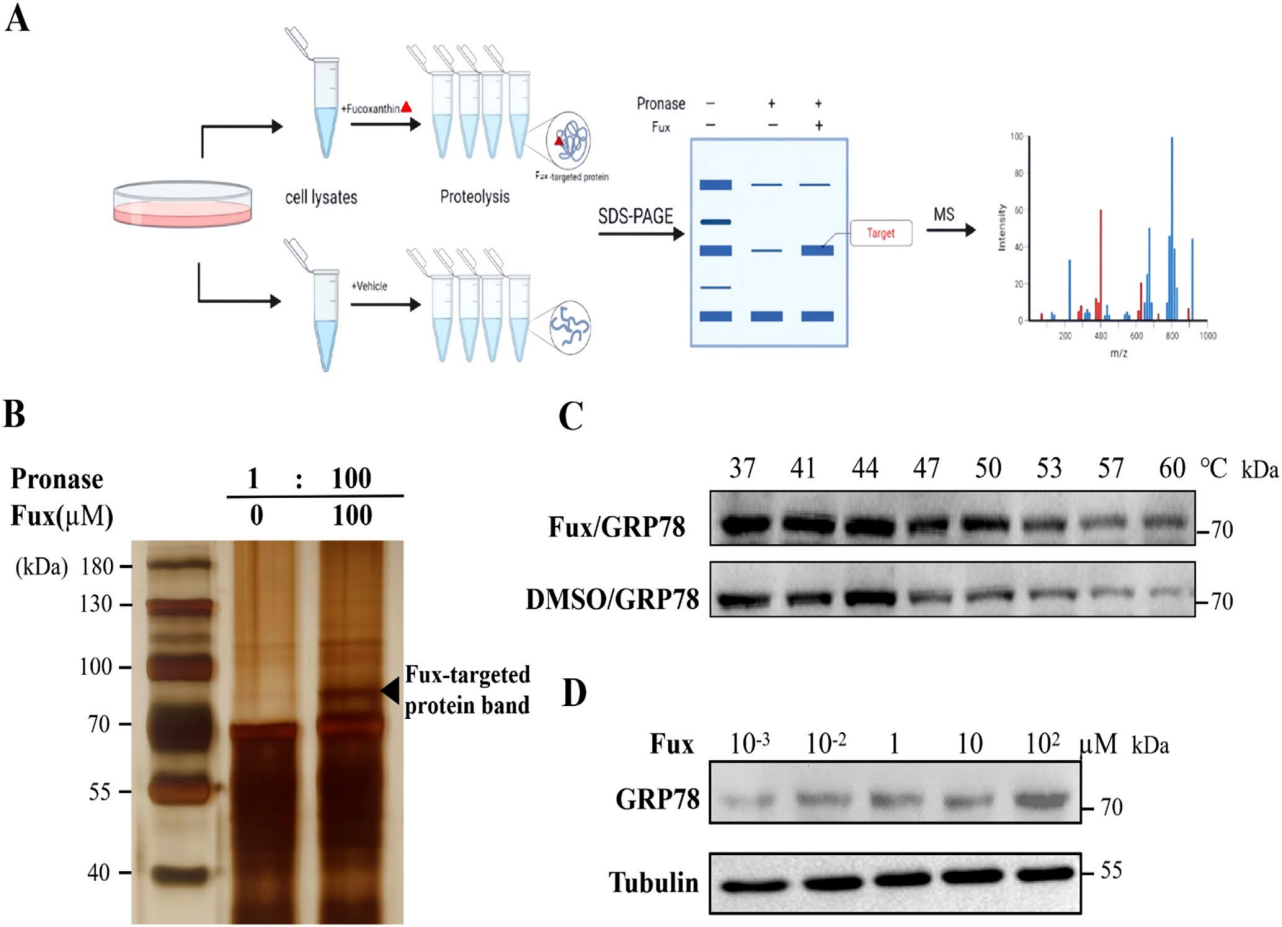
Fucoxanthin directly targets and thermally stabilizes GRP78. (A) Schematic diagram of the drug affinity responsive target stability (DARTS) assay workflow used to identify fucoxanthin‐binding proteins. (B) Representative silver‐stained SDS‐PAGE gel of DARTS assay. HepG2 cell lysates were incubated with fucoxanthin (Fux, 100 μM) or DMSO for 1 h, followed by proteolysis with pronase (1:100 ratio) for 20 min. The black arrow indicates a protected protein band near 78 kDa, identified as GRP78 by MS. (C) Cellular thermal shift assay (CETSA). HepG2 lysates treated with Fux (100 μM) were heated at the indicated temperature gradient (37°C–60°C). Western blots show enhanced thermal stability of GRP78 in the presence of Fux. (D) Isothermal dose–response (ITDR) CETSA. Lysates were treated with increasing concentrations of Fux (10^−3^ to 10^2^ μM) at a fixed temperature (52°C), confirming dose‐dependent stabilization of GRP78.

To establish a cellular model of lipid accumulation, we first evaluated the cytotoxicity of palmitic acid (PA) in HepG2 cells. Treatment with 100 μM PA for 24 h did not cause significant cytotoxicity (cell viability > 90%; Figure S1), and this concentration was therefore selected for subsequent experiments to induce lipid accumulation without inducing massive cell death. In addition, fucoxanthin treatment did not significantly alter body weight or food intake in vivo, further supporting that its effects on lipid metabolism are direct. While our previous work established fucoxanthin as an AMPK activator, its direct upstream target remained unknown. To identify fucoxanthin‐binding proteins, we employed the Drug Affinity Responsive Target Stability (DARTS) assay (Figure 2A). Incubation of HepG2 cell lysates with fucoxanthin (100 μM) followed by limited pronase digestion revealed a distinct protected band near 70–100 kDa (Figure 2B, arrow). Mass spectrometry analysis identified several potential binding partners of fucoxanthin. GRP78 was selected as the top candidate based on its highest peptide coverage and score, as well as its consistency with the bioinformatics prediction. To validate this physical interaction in a cellular context, Cellular Thermal Shift Assay (CETSA) was performed. Fucoxanthin treatment significantly enhanced the thermal stability of GRP78 across a temperature gradient (37°C–60°C) (Figure 2C) and in a concentration‐dependent manner (10^−3^ to 10^2^ μM) at 52°C (Figure 2D), providing compelling biochemical evidence of target engagement.

**FIGURE 3:**
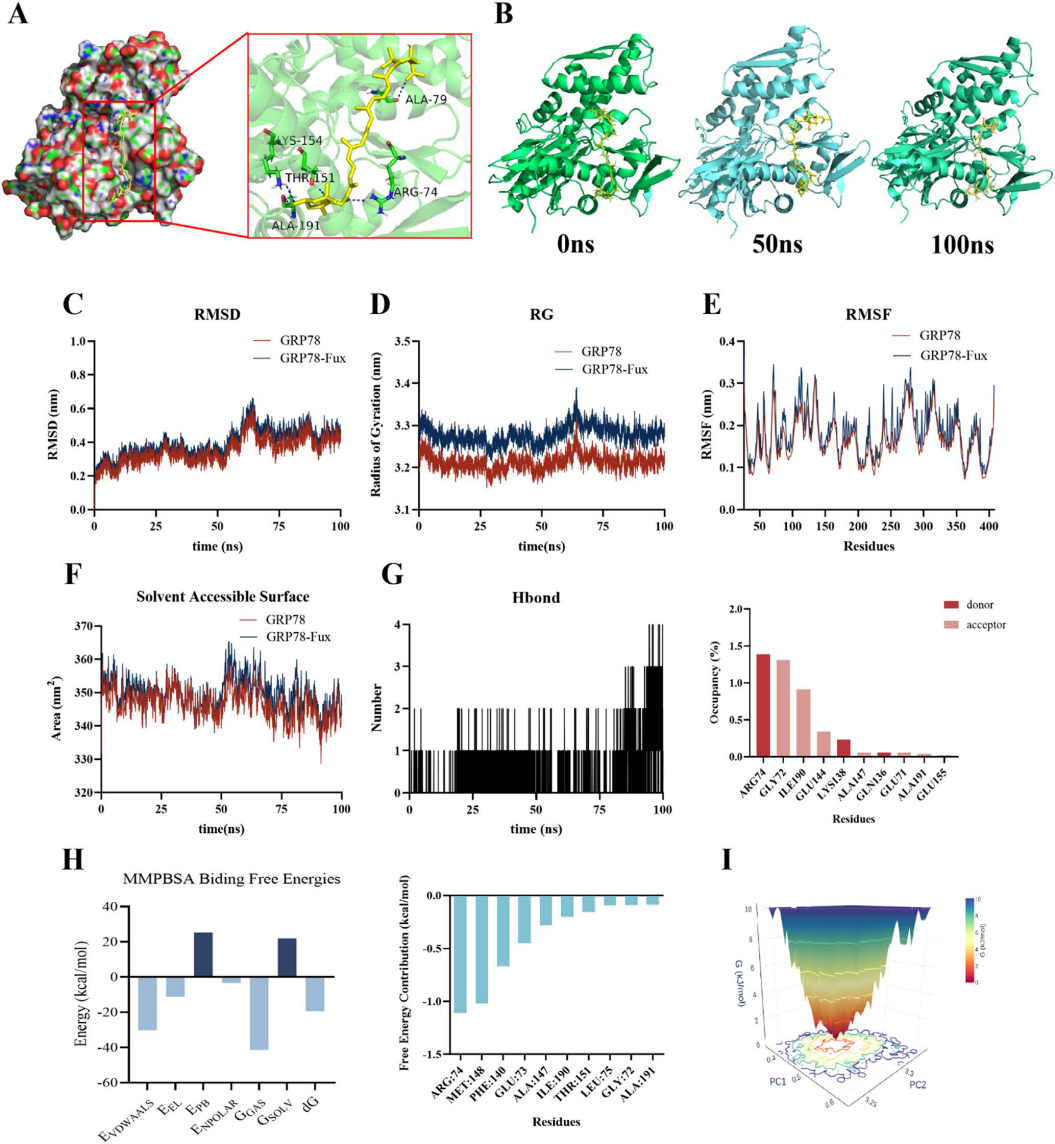
Computational modeling elucidates the binding mode of fucoxanthin to GRP78. (A) Molecular docking pose showing fucoxanthin occupying the binding pocket of GRP78. The interaction is stabilized by hydrogen bonds, particularly with residue ARG74, with a binding affinity of −5.87 kcal/mol. (B) Snapshots from molecular dynamics (MD) simulation at 0 ns, 50 ns, and 100 ns, illustrating the stable retention of the ligand. (C) Root mean square deviation (RMSD) plot of the complex backbone, indicating equilibration after 65 ns. (D) Radius of gyration (Rg) plot showing the compactness of the protein‐ligand complex over time. (E) Root mean square fluctuation (RMSF) analysis of GRP78 residues in the presence (red) or absence (blue) of fucoxanthin. (F) Solvent accessible surface area (SASA) plot showing a decrease over time, suggesting a tighter complex formation. (G) Hydrogen bond analysis revealing the number of H‐bonds throughout the 100 ns simulation. (H) Binding free energy contribution of key residues calculated by MM/PBSA (65–100 ns). ARG74, ILE190, and THR151 are major contributors. (I) Gibbs free energy landscape (FEL) map identifying the most stable conformational state at approximately 65.8 ns.

To elucidate the structural basis of this interaction, we performed molecular docking and molecular dynamics (MD) simulations. Docking analysis predicted that fucoxanthin occupies a specific pocket in GRP78 with a binding energy of −5.87 kcal/mol (Figure 3A). A 100‐ns MD simulation confirmed the stability of this complex (Figure 3B), as evidenced by the equilibrated RMSD (Figure 3C), decreased Radius of Gyration (Figure 3D), and minimal local fluctuations in the binding region (Figure 3E). Mechanistically, hydrogen bond analysis identified ARG74 as a critical residue for maintaining the interaction (Figure 3F). MM/PBSA calculations based on the stable trajectory (65–100 ns) yielded a robust binding free energy of −19.43 kcal/mol, with ARG74, ILE190, and THR151 making the most significant energetic contributions (Figure 3G,H). The Gibbs free energy landscape displayed a single, deep energy minimum, confirming the thermodynamic stability of the fucoxanthin‐GRP78 complex (Figure 3I).

### 3.3 Fucoxanthin Alleviates Hepatic ER Stress and Steatosis in *Ob/Ob* Mice and Palmitic Acid‐Loaded Hepatocytes

**FIGURE 4:**
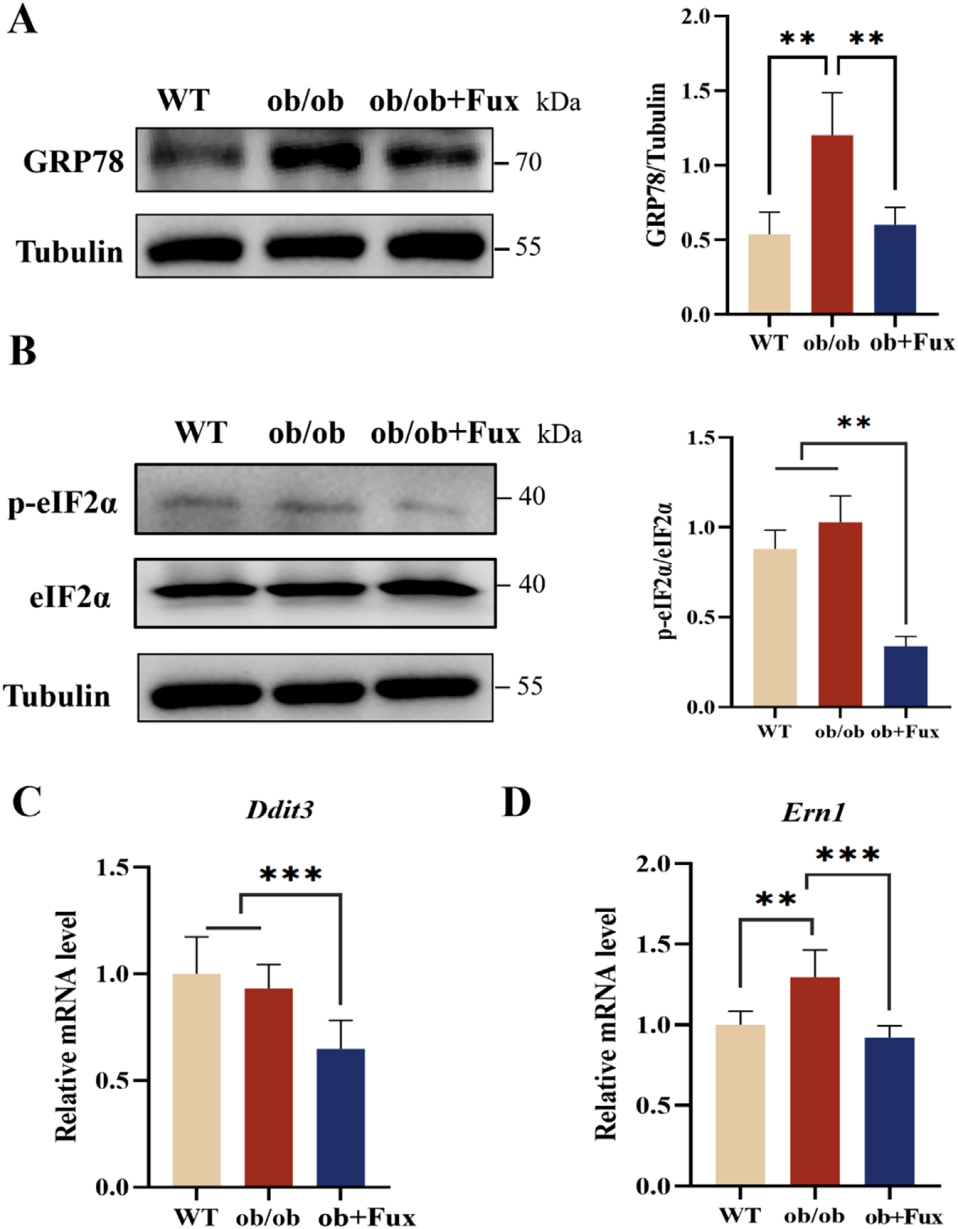
Fucoxanthin alleviates hepatic ER stress in *ob/ob* mic**e**. (A) Representative western blots and quantification of GRP78 protein levels in liver tissues from wild‐type (WT), *ob/ob* model, and fucoxanthin‐treated *ob/ob* mice (0.2% diet). (B) Western blots and quantification of the p‐eIF2α/eIF2α ratio, a marker of UPR activation. (C, D) Relative mRNA expression levels of ER stress markers *Ddit3* (CHOP) and *Ern1* (IRE1) in liver tissues, determined by qRT‐PCR. Data are presented as mean ± SD (*n* = 6–8). *p < 0.05, **p < 0.01, ***p < 0.001.

We next investigated whether targeting GRP78 translates to therapeutic benefits in vivo using leptin‐deficient *ob/ob* mice. Western blot analysis showed that GRP78 protein levels were significantly elevated in *ob/ob* livers compared to WT controls, reflecting chronic ER stress. Fucoxanthin treatment (0.2% dietary supplementation for 8 weeks) remarkably normalized hepatic GRP78 expression (Figure 4A) and significantly reduced the phosphorylation ratio of eIF2α (p‐eIF2α/eIF2α), a hallmark of UPR activation (Figure 4B). Consistently, qRT‐PCR analysis demonstrated that fucoxanthin suppressed the hepatic mRNA expression of key UPR markers, *Ddit3* (CHOP) and *Ern1* (IRE1) (Figure 4C,D).

**FIGURE 5:**
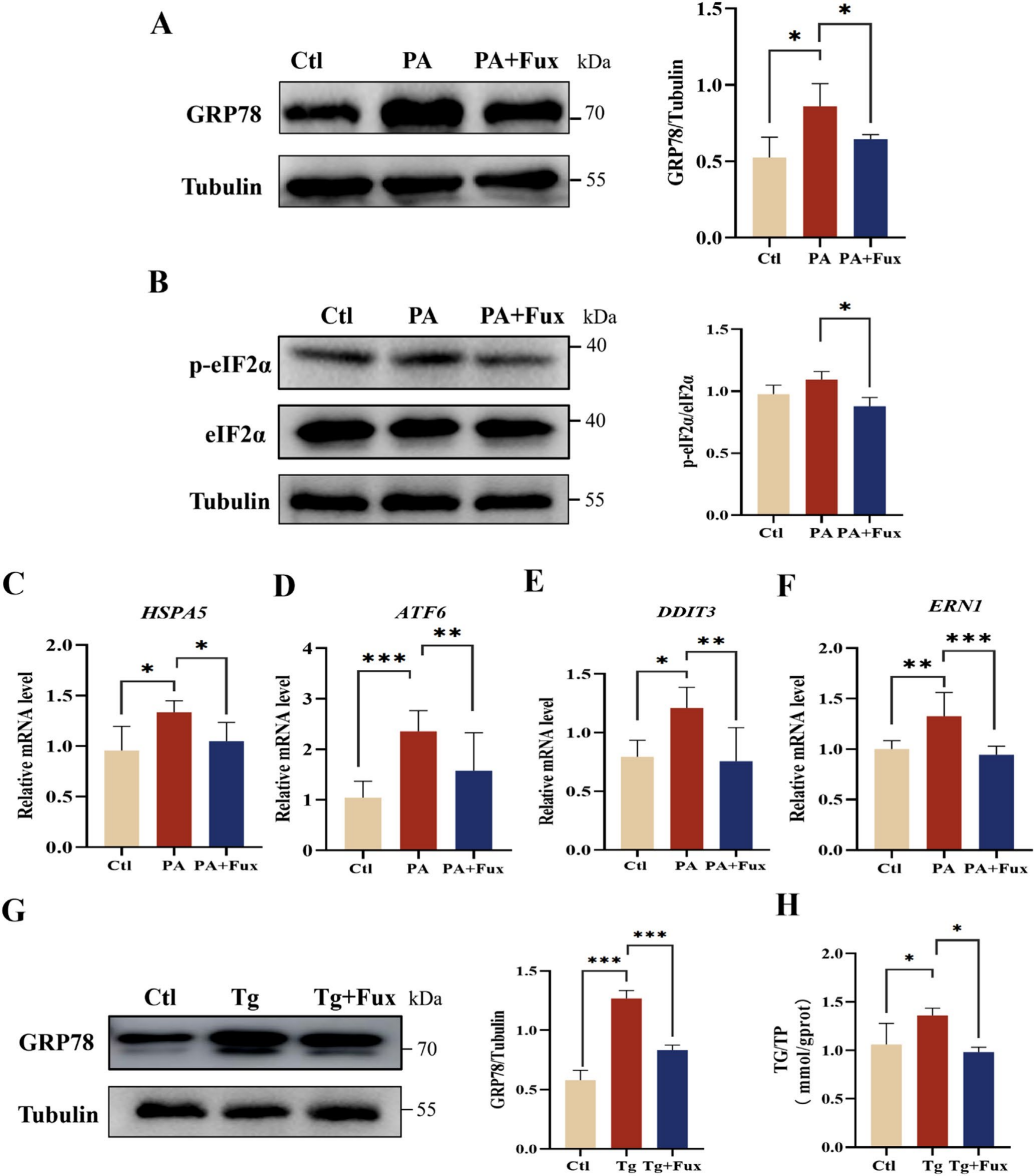
Fucoxanthin mitigates palmitic acid (PA)‐induced ER stress in HepG2 cells. (A, B) Representative western blots and quantification of GRP78 (A) and p‐eIF2α/eIF2α ratio (B) in HepG2 cells treated with PA (100 μM) ± fucoxanthin (Fux) for 24 h. (C–F) Relative mRNA levels of ER stress‐related genes: *HSPA5* (C), *ATF6* (D), *DDIT3* (E), and *ERN1* (F). (G, H) Effect of Fux on thapsigargin (Tg)‐induced ER stress. Cells were treated with Tg (1 μM) ± Fux. (G) Western blots of GRP78 protein expression. (H) Intracellular triglyceride (TG) content normalized to total protein. Data are presented as mean ± SD (*n* = 3). *p < 0.05, **p < 0.01, ***p < 0.001.

To verify these findings in vitro, we utilized a palmitic acid (PA)‐induced HepG2 cell model. PA treatment significantly upregulated GRP78 protein levels and ER stress gene markers (*HSPA5, ATF6, DDIT3, ERN1*). Fucoxanthin intervention reversed these alterations (Figure 5A–F). Furthermore, when cells were challenged with Thapsigargin (Tg), a potent ER stress inducer, fucoxanthin significantly attenuated Tg‐induced GRP78 upregulation (Figure 5G) and intracellular triglyceride accumulation (Figure 5H). These results confirm that fucoxanthin exerts a direct protective effect against ER stress both in vivo and in vitro.

### 3.4 The GRP78‐AMPK Signaling Axis Is Indispensable for the Therapeutic Efficacy of Fucoxanthin

**FIGURE 6:**
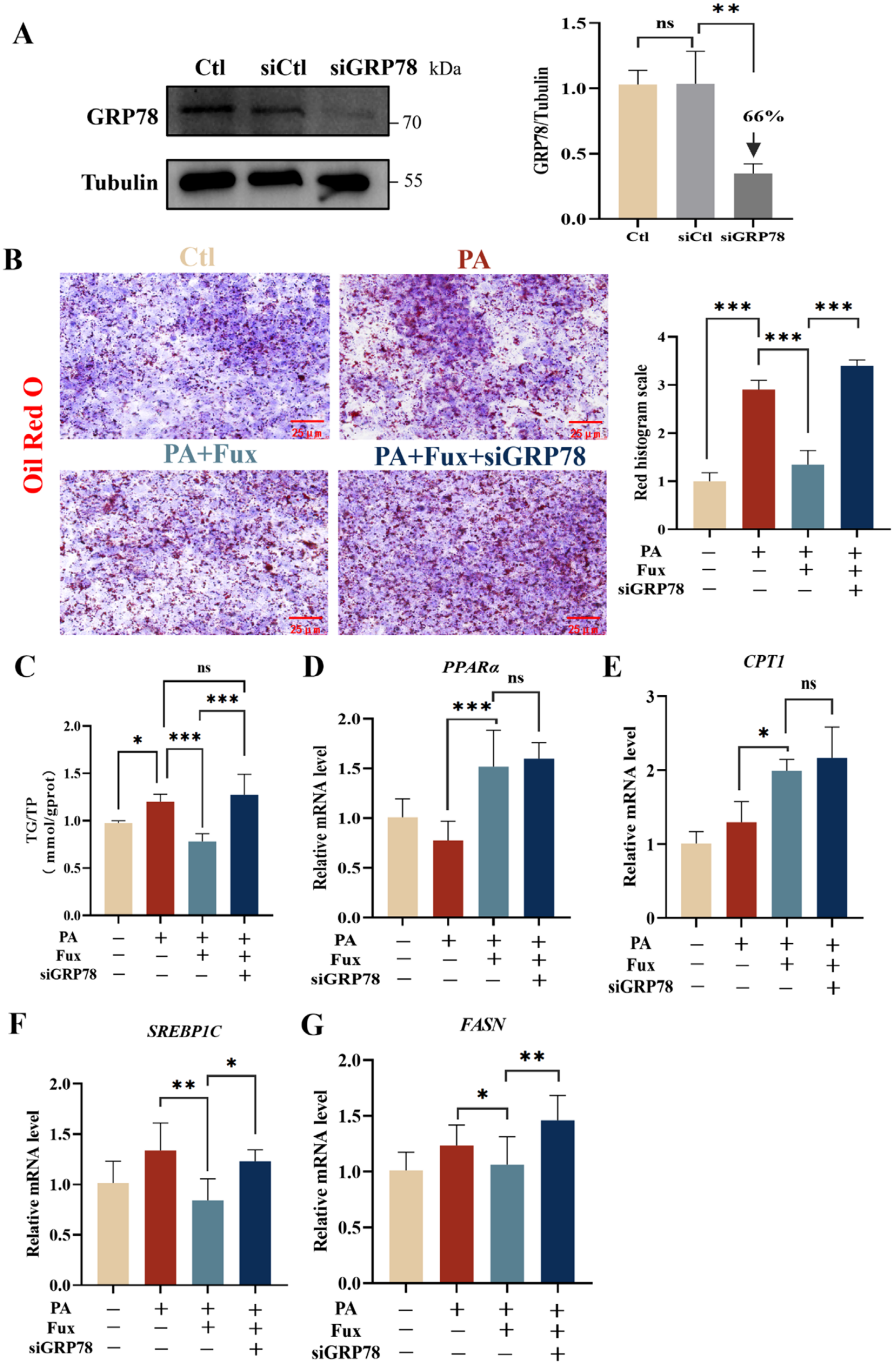
GRP78 knockdown abolishes the lipid‐lowering effect of fucoxanthin. (A) Validation of GRP78 knockdown efficiency by western blot in HepG2 cells. GRP78 protein levels were reduced by approximately 66% in siGRP78‐transfected cells compared to siCtl group. Tubulin served as loading control. (B) Representative Oil Red O staining images (left) and quantification of lipid droplet area (right). GRP78 knockdown reversed the protective effect of Fux against PA‐induced lipid accumulation. (C) Quantification of intracellular TG levels. (D–G) Relative mRNA expression of lipid metabolism genes: Fatty acid oxidation genes *PPARα* (D) and *CPT1* (E); lipogenic genes *SREBP1C* (F) and *FASN* (G). Data are presented as mean ± SD (*n* = 3). *p < 0.05, **p < 0.01, ***p < 0.001, ns, not significant. Scale bar = 25 μm.

Finally, we deciphered the causal link between the fucoxanthin‐GRP78 interaction, AMPK activation, and lipid regulation. We utilized siRNA to knockdown GRP78 expression in HepG2 cells (Figure 6A). In control cells, fucoxanthin effectively reduced PA‐induced lipid accumulation; however, in GRP78‐knockdown cells, this lipid‐lowering effect was completely abolished and lipid deposition was exacerbated (Figure 6B,C). Gene expression analysis showed that GRP78 knockdown abolished the ability of fucoxanthin to downregulate lipogenic genes (*SREBP1C*, *FASN*), while its effect on upregulating fatty acid oxidation genes (*PPARα*, *CPT1*) remained intact. (Figure 6D–G).

**FIGURE 7:**
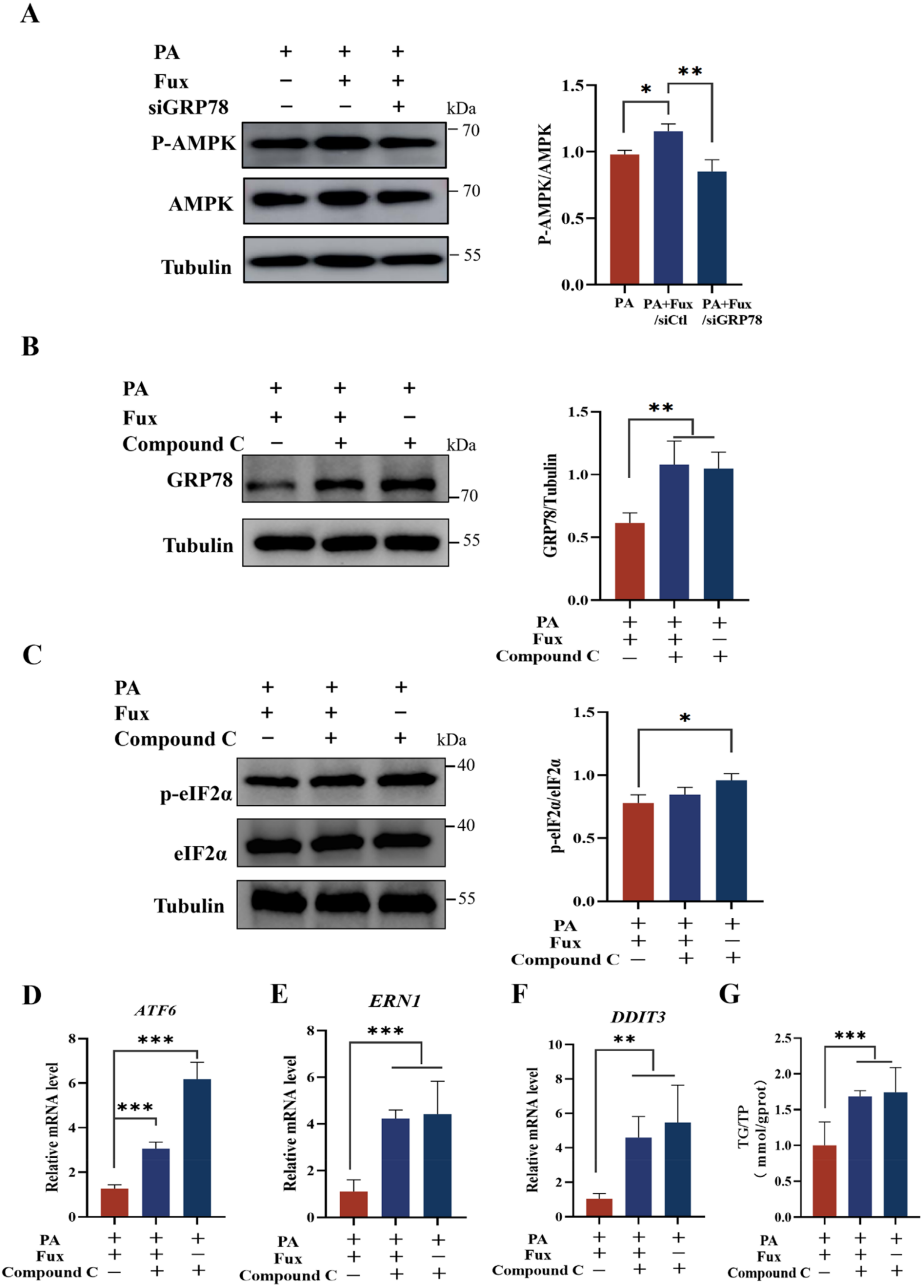
The GRP78‐AMPK axis is essential for the therapeutic efficacy of fucoxanthin. (A) Western blots of p‐AMPK and total AMPK. GRP78 knockdown (siGRP78) prevented Fux‐induced AMPK phosphorylation in PA‐treated cells. (B, C) Effect of AMPK inhibition on ER stress. Cells were pre‐treated with Compound C (10 μM) followed by PA + Fux. Western blots show GRP78 levels (B) and p‐eIF2α/eIF2α ratio (C). (D–F) Relative mRNA expression of ER stress genes: *ATF6* (D), *ERN1* (E), and *DDIT3* (F). Compound C treatment blocked the Fux‐mediated downregulation of these genes. (G) Quantification of intracellular TG levels. AMPK inhibition nullified the lipid‐lowering effect of Fux. Data are presented as mean ± SD (*n* = 3). *p < 0.05, **p < 0.01, ***p < 0.001.

Importantly, we found that GRP78 is required for fucoxanthin‐induced AMPK activation. While fucoxanthin increased p‐AMPK levels in PA‐treated cells, this activation was abrogated in GRP78‐knockdown cells (Figure 7A). Conversely, inhibiting AMPK with Compound C reversed the ability of fucoxanthin to suppress GRP78 expression and ER stress markers (Figure 7B–F) and blocked the reduction in triglyceride content (Figure 7G).

Taken together, these data delineate a novel signaling axis wherein fucoxanthin physically engages GRP78 to trigger AMPK activation, which subsequently alleviates ER stress and inhibits lipogenesis. This confirms that GRP78 serves as the indispensable upstream initiator governing the therapeutic efficacy of fucoxanthin in MASLD.

## 4 Discussion

The escalating prevalence of MASLD necessitates the discovery of novel therapeutic agents that can simultaneously tackle hepatic steatosis and organelle dysfunction (Yang et al. 2025). While fucoxanthin has shown promise as a metabolic regulator, its precise molecular targets within the hepatocyte have remained elusive. In this study, we integrated multi‐omics analysis, chemoproteomics, and molecular simulation to provide the first evidence that GRP78 is a direct, functional target of fucoxanthin. We demonstrate that fucoxanthin physically engages the ER chaperone GRP78, thereby activating the AMPK signaling axis to restore ER homeostasis and reprogram lipid metabolism. This target‐driven mechanism distinguishes fucoxanthin from non‐specific antioxidants and positions the GRP78‐AMPK axis as a pivotal therapeutic interface for MASLD.

The identification of GRP78 as a fucoxanthin target is particularly significant given the central role of ER stress in MASLD progression. Under lipotoxic conditions, the adaptive capacity of the ER is overwhelmed, leading to a “maladaptive UPR” that exacerbates lipogenesis and inflammation (Lebeaupin et al. 2018; Shang et al. 2025). GRP78 sits at the apex of this regulatory network, gating the activation of IRE1, PERK, and ATF6. Our data show that fucoxanthin does not merely suppress downstream ER stress markers (e.g., CHOP, p‐eIF2α) but acts at the source by stabilizing GRP78 itself. The residue ARG74, identified as the key binding site for fucoxanthin, is located in the Nucleotide‐Binding Domain (NBD) of GRP78. Our docking results suggested that fucoxanthin binding might induce a conformational change in the NBD. Interestingly, our experimental data showed that fucoxanthin does not inhibit ATPase activity, suggesting it acts as a modulator rather than a competitive inhibitor. Notably, molecular dynamics simulations revealed that fucoxanthin binds to a specific pocket involving the ARG74 residue of GRP78. Unlike classical chemical chaperones (e.g., TUDCA/PBA) that function primarily as solvent stabilizers (Alotaibi and Alkhammash 2025; Freitas et al. 2023), fucoxanthin appears to act as a specific ligand that modulates GRP78 conformation. This “ligand‐like” behavior suggests a more potent and specific mode of action in rectifying ER dysfunction.

A pivotal mechanistically relevant question raised by our findings is the spatial coordination between the ER‐resident GRP78 and the primarily cytosolic AMPK. Although GRP78 (BiP) is traditionally localized within the ER lumen, our results unequivocally demonstrate that GRP78 knockdown abrogates fucoxanthin‐induced AMPK phosphorylation, establishing GRP78 as an essential upstream regulator in this signaling axis. To reconcile this topological separation, we propose a “conformational signaling” model facilitated by the dynamic nature of GRP78. Under metabolic stress, GRP78 is known to interact with transmembrane ER sensors or even translocate to the cytosol and cell surface (Makio et al. 2024). We hypothesize that fucoxanthin binding to the NBD of GRP78 induces a specific conformational change that triggers downstream signaling through two potential non‐exclusive mechanisms. One possible pathway involves the calcium–CaMKKβ axis. GRP78 acts as a major gatekeeper of ER calcium (Ca^2+^) storage (Daverkausen‐Fischer and Pröls 2022). Fucoxanthin binding may modulate GRP78's interaction with ER calcium‐release channels (such as IP3R), leading to a controlled “leakage” of Ca^2+^ into the cytosol. This localized increase in cytosolic Ca^2+^ would subsequently activate CaMKKβ, a canonical upstream kinase that phosphorylates AMPK at Thr172 (McAloon et al. 2024). Alternatively, GRP78 may serve as a molecular scaffold that interacts with ER‐localized transmembrane proteins (e.g., PERK or IRE1). Fucoxanthin might stabilize a GRP78‐transmembrane complex that recruits or releases LKB1 from the ER periphery into the cytosol, thereby facilitating its interaction with the AMPK complex (Marcondes‐de‐Castro et al. 2023). Importantly, our observation that fucoxanthin does not inhibit GRP78's ATPase activity suggests that it modulates, rather than blocks, the chaperone's functional cycle, preserving its essential physiological roles while enhancing its stress‐resolving capacity.

This study further elucidates how fucoxanthin breaks the “lipotoxicity‐ER stress vicious cycle.” In MASLD, lipid overload triggers ER stress, which in turn promotes de novo lipogenesis via SREBP‐1c (SREBF‐1c) activation, creating a positive feedback loop (Ke et al. 2025; Li et al. 2023). We found that the GRP78‐AMPK axis exerts a dual effect: (1) “Metabolic Rescue”: Activated AMPK phosphorylates ACC and downregulates SREBP‐1c/FASN, cutting off lipid synthesis and promoting fatty acid oxidation (PPARα/CPT1); (2) “ER Reinforcement”: By reducing the lipid load on the ER membrane and potentially modulating UPR signaling directly, GRP78 stabilization restores protein folding capacity. When we blocked AMPK with Compound C, the ability of fucoxanthin to alleviate ER stress was lost, indicating that metabolic improvement is a prerequisite for, or at least tightly coupled with, the resolution of ER stress. This synergistic “dual‐hit” on both metabolism and stress response underpins the potent efficacy of fucoxanthin.

Despite these promising findings, some limitations warrant consideration. While we confirmed the fucoxanthin‐GRP78 interaction using DARTS and CETSA, future structural biology studies (e.g., X‐ray crystallography) would be valuable to visualize the precise binding interface. Additionally, while we utilized *ob/ob* mice and cell models, verifying this mechanism in liver‐specific GRP78 knockout mice would further strengthen the causal conclusions in vivo. Nevertheless, our findings have immediate translational implications. They suggest that dietary supplementation with fucoxanthin‐rich seaweeds or purified nutraceuticals could serve as an effective strategy for MASLD prevention. Furthermore, the GRP78‐binding pocket identified here could serve as a template for designing high‐affinity synthetic ligands to treat ER stress‐related metabolic diseases.

For future clinical translation, achieving this dose would indeed require concentrated fucoxanthin supplements or bioavailability‐enhanced formulations. Notably, fucoxanthin has demonstrated a favorable safety profile, showing no toxic side effects or genotoxicity/mutagenicity (Zhang et al. 2024), and advanced nanotechnologies enhance its bioactivity and therapeutic performance (Wang et al. 2025). These findings support its potential for further clinical development, though higher doses would necessitate additional safety evaluation. The translational feasibility of our findings will depend on establishing the minimum effective dose in humans and developing formulations with improved oral bioavailability.

In conclusion, this study unravels a novel molecular mechanism wherein fucoxanthin acts as a GRP78‐targeting AMPK activator. By directly binding to GRP78, fucoxanthin orchestrates a therapeutic response that resolves ER stress and corrects lipid metabolic dysfunction. These findings not only clarify the pharmacological basis of fucoxanthin but also highlight the GRP78‐AMPK axis as a druggable target for nutritional and pharmaceutical interventions in MASLD.

## Author Contributions

**Cheng Wang:** project administration, resources. **Jingui Zhang:** resources, funding acquisition. **Zhengshun Wen:** formal analysis, visualization. **Liang Hong:** funding acquisition, writing – review and editing, project administration, supervision, resources. **Binbin Chen:** supervision, resources, project administration. **Haibin Tong:** writing – review and editing, funding acquisition, resources, project administration, supervision. **Ruifen Zhang:** software, investigation, data curation, validation, formal analysis. **Yu Wu:** software, methodology, validation. **Lin Zhang:** writing – original draft, conceptualization, methodology, visualization, formal analysis, software, data curation. **Chao Wang:** visualization, project administration, resources.

## Funding

This work was financially supported by the Chronic Disease Management Research Project of the National Health Commission Capacity Building and Continuing Education Center (No. GWJJMB202510024127); the Key Project at Central Government Level: The Ability Establishment of Sustainable Use for Valuable Chinese Medicine Resources (No. 2060302); the Major Science and Technology Project of New Agricultural Varieties Breeding in Wenzhou (No. ZX2024003‐4); the Zhejiang Province “Three Rural Nine Parties” Science and Technology Collaboration Program (No. 2025SNJF041); and the Hainan Provincial Natural Science Foundation of China (No. 822QN453).

## Conflicts of Interest

The authors declare no conflicts of interest.

## Supporting information


**Figure S1:** Effect of palmitic acid (PA) on the viability of HepG2 cells. HepG2 cells were treated with increasing concentrations of PA (as indicated) for 24 h. Cell viability was determined using the CCK‐8 assay. Data are presented as mean ± SD (*n* = 3). No significant cytotoxicity was observed at 100 μM PA compared to the control group (viability > 90%). Statistical analysis was performed by one‐way ANOVA followed by Dunnett's test; ns, not significant.

## Data Availability

The data that support the findings of this study are available from the corresponding author upon reasonable request.
