# Supplementary material for: Fucoxanthin Ameliorates MASLD by Directly Targeting GRP78 to Restore ER Homeostasis and Activate AMPK Signaling
Source: Food Sci Nutr. 2026 Apr 30;14(5):e71813. doi: 10.1002/fsn3.71813 (PMC13129686; doi:10.1002/fsn3.71813)
Supplement: Supplementary file 1 — Figure S1: Effect of palmitic acid (PA) on the viability of HepG2 cells. HepG2 cells were treated with increasing concentrations of PA (as indicated) for 24 h. Cell viability was determined using the CCK‐8 assay. Data are presented as mean ± SD (n = 3). No significant cytotoxicity was observed at 100 μM PA compared to the control group (viability > 90%). Statistical analysis was performed by one‐way ANOVA followed by Dunnett's test; ns, not significant. [file FSN3-14-e71813-s001.doc]

**Supplementary Material**

**Supplementary Methods**

**Cell Viability Assay**

HepG2 cells were seeded in 96-well plates at a density of 1 × 10⁴ cells per well and cultured overnight. Cells were then treated with various concentrations of palmitic acid (PA: 50, 100, 200, 400 μM) for 24 h. Cell viability was measured using the Cell Counting Kit-8 (CCK-8; Dojindo, Japan) according to the manufacturer's instructions. Absorbance was read at 450 nm using a microplate reader. All experiments were performed in triplicate.


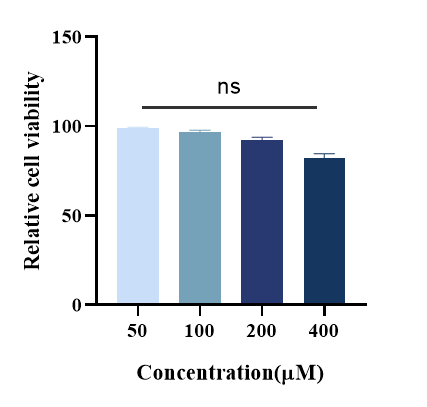


**Supplementary Figure 1. Effect of palmitic acid (PA) on the** **viability of HepG2 cells.** HepG2 cells were treated with increasing concentrations of PA (as indicated) for 24 h. Cell viability was determined using the CCK-8 assay. Data are presented as mean ± SD (n = 3). No significant cytotoxicity was observed at 100 μM PA compared to the control group (viability > 90%). Statistical analysis was performed by one-way ANOVA followed by Dunnett's test; ns, not significant.
